# Fibroblast Growth Factor 23 and Sarcopenia in Maintenance Haemodialysis Population

**DOI:** 10.1002/jcsm.13848

**Published:** 2025-06-04

**Authors:** Limy Wong, Rachel Kenny, Jenny Y. Y. Ooi, Yung Shing Tsang, Emily Schembri, Lawrence P. McMahon

**Affiliations:** ^1^ Department of Renal Medicine Monash University Eastern Health Clinical School Victoria Australia; ^2^ Department of Renal Medicine Eastern Health Victoria Australia; ^3^ Eastern Health Clinical School Monash University Victoria Australia

**Keywords:** dialysis, FGF23, muscle strength, sarcopenia

## Abstract

**Background:**

Sarcopenia is defined as the loss of muscle mass, strength, and/or performance. It is strongly associated with all‐cause mortality. Fibroblast growth factor 23 (FGF23) is markedly elevated in patients with chronic kidney disease, especially those receiving maintenance dialysis. FGF23 has previously been shown to have a direct role in cardiac dysfunction mediated through left ventricular hypertrophy. However, its role in the development of (or protection from) sarcopenia is uncertain. This study is aimed at determining the relationship between FGF23 and muscle‐related parameters and to assess the effect of FGF23 on skeletal muscle myoblasts.

**Methods:**

A single centre, cross‐sectional study examining maintenance haemodialysis patients was conducted. Sarcopenia was defined in accordance with the revised European Working Group on Sarcopenia in Older People and the Asian Working Group for Sarcopenia criteria. Clinical assessment methods included bioelectrical impedance analysis, anthropometric measurement, handgrip strength and physical performance appraisal. Both intact FGF23, which is biologically active, and the inactive C‐terminal FGF23 were measured using enzyme‐linked immunosorbent assays. The direct effects of FGF23 on skeletal muscle myoblast proliferation and myogenic differentiation were assessed using an in vitro culture system. Linear and logistic regression analyses were performed to examine the associations between FGF23 and muscle‐related parameters and sarcopenia, respectively.

**Results:**

Eighty‐one patients were included with a median age of 75 years (interquartile range 67–80), and 63% were male. Log‐transformed serum FGF23 correlated positively with handgrip strength (*r* = 0.27, *p* = 0.01, 95% confidence interval (CI) 0.06–0.46) and calf circumference (*r* = 0.27, *p* = 0.01, 95% CI 0.06–0.46), and in multiple regression analyses, it was found to be a significant independent predictor of both handgrip strength (beta = 5.39, 95% CI 2.07–8.72) and sarcopenia (odds ratio = 0.14, 95% CI 0.02–0.75). FGF23 was found to promote myoblast proliferation but attenuate myogenic differentiation. At 48 h of differentiation, the expressions of *MyoG* and *MyoD* were significantly lower in cells treated with FGF23 than the control. The fusion index and myotube diameters were reduced on Day 7 of differentiation in FGF23‐treated cells compared to the control.

**Conclusions:**

Higher serum FGF23 levels were associated with stronger handgrip strength and lower odds of having sarcopenia in maintenance haemodialysis patients. Our findings suggest that supraphysiological levels of FGF23 might play a role in muscle regeneration by promoting myoblast proliferation but repressing myogenic differentiation to support the expansion of the proliferative pool. FGF23 could potentially serve as a serum biomarker for muscle health in dialysis populations.

## 1 Introduction

Sarcopenia is defined as loss of muscle mass, strength and/or performance that worsens with age. It can arise in association with systemic illnesses, particularly those that invoke an inflammatory process, such as chronic kidney disease (CKD) [1]. The reported prevalence of sarcopenia in CKD has varied markedly, ranging from 3.8% to 98.5% in maintenance dialysis patients, depending on the definitions and diagnostic thresholds used [2, 3, 4, 5, 6, 7]. A recent systematic review and meta‐analysis involving more than 40 000 patients at different stages of CKD across 25 countries reported a global prevalence of sarcopenia of 24.5%, with higher rates of severe sarcopenia in patients on dialysis [8]. Sarcopenia is associated with a higher mortality in CKD patients [9]. Numerous risk factors have been identified to contribute to the development of sarcopenia. These include ageing, immunosenescence, hormonal imbalance, sedentary/inactive lifestyle, and poor nutritional status. In CKD, additional factors include metabolic acidosis, accumulation of uraemic toxins, and a chronic state of catabolism in maintenance haemodialysis patients [10].

We previously investigated the clinical significance of low skeletal muscle mass in kidney transplant recipients and found that both serum phosphate and parathyroid hormone (PTH) levels were independently associated with low skeletal muscle mass [11]. The temporal changes of disordered mineral metabolism in CKD patients are well‐established, with a progressive rise in circulating fibroblast growth factor (FGF) 23 (FGF23) early in the course of CKD, followed by secondary hyperparathyroidism and hyperphosphataemia in the late stages [12]. FGF23 is a bone‐derived phosphaturic hormone, whose action is dependent upon several factors, including fibroblast growth factor receptors (FGFRs) and, in certain cell types (kidney and parathyroid glands), α‐klotho coreceptors. Serum levels of FGF23 can increase up to 1000‐fold above normal in maintenance dialysis patients [13]. Clinically, elevated FGF23 has been associated with an increased risk of major cardiovascular events and mortality [14, 15, 16], and in vitro has been shown to induce pathological hypertrophy of isolated cardiomyocytes in the absence of α‐klotho, producing left ventricular hypertrophy in a murine model following intraventricular or intravenous FGF23 injection [17].

FGFRs are known to be expressed during skeletal muscle development and myogenesis. Given the direct effects of FGF23 on cardiac muscle, there may be a connection between FGF23 and skeletal muscle function. Previous studies demonstrated positive associations between plasma FGF23 and muscle mass indices [18]. Furthermore, targeted ablation of FGF23 results in skeletal muscle atrophy in mice [19]. However, the discrete effect of FGF23 on skeletal muscle function remains unclear. We hypothesised that supraphysiological levels of FGF23 would be associated with skeletal muscle growth and regeneration. We conducted this study to determine the prevalence of sarcopenia and to investigate the relationship between FGF23 and sarcopenia in a well‐characterised cohort of maintenance dialysis patients. We also assessed the direct effect of FGF23 on skeletal myoblasts using an in vitro culture system.

## 2 Methods

### 2.1 Human Study Population

This cross‐sectional study analysed data from SPARSE (Sarcopenia in Patients with Chronic Kidney Disease), a prospective observational study conducted at a large, tertiary‐referral academic renal service in metropolitan Australia. The study was approved by the local Human Research Ethics Committee (reference number E20‐025‐67 026). Full written informed consent was obtained from all study participants. Maintenance dialysis patients aged 50 years or over were eligible for inclusion. All participants were recruited to the study when attending the health service for routine healthcare encounters (e.g., outpatient clinic appointments or haemodialysis sessions). Alongside completion of study questionnaires, demographic and medical history data were concurrently collected. Anthropometric measurements were obtained including body mass index (BMI), midupper arm circumference (MUAC) and calf circumference (CC).

### 2.2 Assessment of Sarcopenia

Sarcopenia was defined according to both the European Working Group on Sarcopenia in Older People (EWGSOP 2019) [1] and Asian Working Group for Sarcopenia (AWGS 2019) [20] definition criteria as a substantial proportion of the study participants were Asian. Three diagnostic categories were defined: the presence of low muscle strength alone (probable sarcopenia), low strength and reduced muscle mass (sarcopenia), and low physical performance (severe sarcopenia; see Supplementary Methods for further details). Appendicular skeletal muscle index (ASMI, in kilograms per square meter) was derived from the appendicular muscle mass (in kilogram) divided by height squared (in square meters). Muscle mass was measured by bioelectrical impedance analysis (BIA) using a Tanita MC780 Professional Body Composition Scale after a time interval of 15–20 min following the end of the midweek haemodialysis session to reflect a dry weight status. Muscle strength was measured using a Jamar J00105 hydraulic hand dynamometer. Physical performance was assessed using the timed up‐and‐go and chair‐to‐stand tests.

### 2.3 Blood Sampling and Laboratory Measurements

Routine standard care blood tests and additional serum samples were collected, centrifuged, and stored at −80°C. Blood samples from haemodialysis patients were obtained predialysis from the arterio–venous fistula or a tunnelled dialysis catheter during the midweek dialysis session. Biochemical analyses, including full blood count, serum creatinine and electrolytes, liver function, C‐reactive protein (CRP), iron studies, 25(OH)‐vitamin D, glycated haemoglobin and PTH were performed using standard laboratory techniques. Both intact FGF23 (iFGF23), which is biologically active, and the inactive C‐terminal FGF23 (cFGF23) were measured in duplicate by enzyme‐linked immunosorbent assays (ELISA) with a human FGF23 immunometric assay according to the manufacturer's instructions and reported as picograms per milliliter (Merck Millipore Immunoassay, Massachusetts, United States) and RU/mL (Immutopics, San Clemente, United States) respectively. The intra‐assay coefficient of variation (CV) using duplicates from the patient cohort was 5.14% for iFGF23 and 5.42% for cFGF23.

Both indoxyl sulphate (IS) and p‐cresyl sulphate (PCS) were measured as previously described by Calaf et al. with minor modifications [21, 22]. Briefly, 300 mL of 100% ethanol was added to 100 mL of serum, saturated with 100 mg of sodium chloride, followed by adding 700 mL of mobile phase A and centrifuged for 10 min at 10000 g. Samples were then assayed on a Shimadzu ultraperformance liquid chromatography system, with a Merck Lichrospher 60 Select B 5 μm, 125 × 4 mm reverse‐phase column. Mobile Phase A was 20 mM sodium dihydrogen phosphate and 5 mM tetrabutylammonium iodide in water, and Mobile Phase B was acetonitrile. All samples were run in duplicates; IS and PCS were quantified on a fluorescence detector at excitation: emission wavelengths of 278:348 nm and 260:285 nm, eluting at 7.74 and 11.6 min, respectively.

### 2.4 Cell Culture

Normal adult human skeletal muscle myoblasts (HSMMs) (Lonza Walkersville, United States) were plated at 5000–8000 cells/cm^2^ and cultured in growth medium (SkGM‐2 BulletKit Medium, Lonza) at 37° in a controlled humidified 5% CO_2_ atmosphere. To induce differentiation into myotubes, upon 70%–80% confluency, growth medium was switched to differentiation medium (DMEM:F‐12, Lonza; supplemented with 2% horse serum and 1% penicillin–streptomycin). Cells were cultured for 5–7 days into multinucleated myotubes, with media changes occurring every other day.

### 2.5 RNA Isolation and Real‐Time Polymerase Chain Reaction (RT‐PCR)

Total RNA was extracted from skeletal muscle myoblasts and differentiating skeletal muscle myoblasts, treated with 100 ng/mL recombinant human fibroblast growth factor 2 (FGF2) (previously also known as basic FGF; R&D Systems) as a positive control, 100 ng/mL recombinant human FGF23 (R&D Systems) or uraemic toxins (IS 1 mM, Sigma Aldrich and PCS 0.2 mM, APExBio) as a negative control, using the RNeasy mini extraction kit (Qiagen) according to the manufacturer's instructions. The RNA yield and quality were assessed using a NanoDrop Lite spectrophotometer (Thermo Fisher Scientific). Complementary DNA (cDNA) was generated using the Superscript III First‐Strand Synthesis SuperMix (Thermo Fisher Scientific) with a total RNA input of 150 ng. RT‐PCR was carried out using TaqMan Gene Expression Assays (Thermo Fisher Scientific; see Supporting Information for details) with the CFX Opus 384 Real‐Time PCR system (BIO‐RAD). The amplification plots for the entire plate were viewed, and comparative cycle threshold (C_T_) values were generated for relative quantification of gene expression analysis, which was performed using the 2^−∆CT^ or 2^−∆∆CT^ methods, using β‐actin as the housekeeping gene.

### 2.6 Cell Proliferation

For proliferation assays, undifferentiated human myoblasts were treated with FGF23 (100 ng/mL), FGF2 (100 ng/mL) as a positive control, and uraemic toxins (IS 1 mM and PCS 0.2 mM) as negative controls at 48 and 72 h. Cell proliferation was measured in 96‐well plates using the BrdU (5‐bromo‐2′‐deoxyuridine) assay (Roche). During the BrdU assay, BrdU is incorporated into replicating DNA and can be detected using anti‐BrdU antibodies.

### 2.7 Fluorescence Microscopy and Morphometry

After 7 days of culturing in a differentiation medium, myotubes were fixed with 10% neutral buffered formalin for 10 min at room temperature (RT). Cells were treated with 0.1% polyethylene glycol tert‐octylphenyl ether (Triton X‐100; Sigma‐Aldrich) for 30 min at RT and blocked with 1% bovine serum albumin (BSA) in phosphate‐buffered saline (PBS) for 1 h at RT. Cells were then incubated with unconjugated primary antibody against myosin (1:200 dilution in blocking solution; PA5–31466, Thermo Fisher) overnight at 4°C and Alexa Fluor 594 conjugated goat anti‐rabbit IgG secondary body (1:200 dilution in blocking solution; Thermo Fisher) along with 4′,6‐diamidino‐2‐phenylindole (DAPI in 1:1000 dilution) and phalloidin FITC reagent (1:40 dilution; Abcam) for 1 h at RT. Cells were mounted with Prolong gold antifade mountant (Thermo Fisher) for microscopic observation using a Leica AF6000KLX microscope system. To analyse myotube formation, fusion index and myotube diameters were measured from three randomly chosen fields per well. The myotube fusion index was calculated as the number of nuclei localised inside myosin‐positive myotubes divided by the total number of nuclei present in a field of view, whilst the myotube diameters were calculated as the average from three to six independent measurements per myosin‐positive myotube.

### 2.8 Statistical Analysis

Baseline characteristics were compared between sarcopenic and nonsarcopenic maintenance haemodialysis patients. The normality of distribution of continuous variables was tested by Shapiro–Wilk test and Kolmogorov–Smirnov test. Continuous variables with normal distribution were expressed as mean (standard deviation (SD)), whilst nonnormally distributed variables were presented as median (interquartile rage (IQR)). Categorical variables were presented as frequency (percentages). Means of two continuous normally distributed variables were compared by independent samples Student's *t*‐test. Mann–Whitney U test and Kruskal–Wallis test were used, respectively, to compare means of two and three groups of variables that were not normally distributed. The frequencies of categorical variables were compared using the Pearson *χ* ^2^ test. Correlation between continuous variables was analysed using the Pearson correlation coefficients if variables were normally distributed or with Spearman's rank correlation coefficients otherwise. Linear regression analyses were performed to examine the association between FGF23 and different parameters of sarcopenia (handgrip strength and CC) and logistic regression to analyse the odds of having sarcopenia. FGF23 was modelled on a continuous scale after natural log transformation was used to achieve normality. A sequence of multivariable models was evaluated for each analysis. Model 1 adjusted for age, gender, Charlson Comorbidity Index (CCI), BMI, serum creatinine, calcium, phosphate, PTH and 25(OH)‐vitamin D. Model 2 included Model 1 parameters, in addition to serum albumin, CRP and IS. In vitro studies were analysed with either a two‐tailed unpaired *t*‐test when measuring comparisons between two groups or a Kruskal–Wallis test or one‐way ANOVA when comparing between more than two groups. Data were presented as mean ± SEM. All analyses were analysed using GraphPad Prism Version 10.1.1 for macOS, and *p* values of less than 0.05 were considered statistically significant.

## 3 Results

Sarcopenia is prevalent in maintenance haemodialysis patients. Table 1 presents all participants stratified according to the presence or absence of sarcopenia. The median age of the study cohort was 75 (IQR 67–80) years, and 63% were male. Sixty (74%) dialysis patients were probable sarcopenic, sarcopenic or severely sarcopenic. These patients were older, more likely to be using walking aids when mobilising, had a higher CCI, lower handgrip strength, prolonged timed‐up‐and‐go and chair‐to‐stand assessments. No differences between sarcopenic and nonsarcopenic patients were observed in BMI, MUAC, CC or ASMI.

**TABLE 1:** Demographics, laboratory and functional capacity of the study population.

Parameters	All (*n* = 81)	No sarcopenia (*n* = 21)	Sarcopenia (*n* = 60)	*p*
Demographics				
Age in years (mean, IQR)	74.5 (67.3–80.1)	68.6 (59.1–76.2)	75.9 (70.6–80.4)	< 0.01
Sex (*n*, %)				0.30
Male	51 (63.0)	11 (52.4)	40 (66.7)	
Female	30 (37.0)	10 (47.6)	20 (33.3)	
Ethnicity (*n*, %)				0.81
Caucasian	58 (71.6)	14 (66.7)	44 (73.3)	
Asian	20 (24.7)	6 (28.6)	14 (23.3)	
Other	3 (3.7)	1 (4.7)	2 (3.4)	
Smoking status (*n*, %)				0.57
Current smoker	4 (4.9)	2 (9.5)	2 (3.3)	
Previous smoker	31 (38.3)	9 (42.9)	22 (36.7)	
Living status (*n*, %)				0.64
Alone	14 (17.3)	3 (14.3)	11 (18.3)	
With family/friends	63 (77.8)	18 (85.7)	45 (75.0)	
Residential aged care facility	4 (4.9)	0 (0)	4 (6.7)	
Primary kidney disease (*n*, %)				0.40
Diabetes/hypertension	48 (59.3)	10 (47.6)	38 (63.3)	
Glomerulonephritis/cystic kidney	24 (29.6)	8 (38.1)	16 (26.7)	
Others (reflux, obstructive and myeloma)	9 (11.1)	3 (14.3)	6 (10.0)	
Charlson Comorbidity Index (mean, IQR)	8.0 (6.0–9.0)	6.0 (4.0–8.0)	8.0 (7.0–9.0)	< 0.01
Mobility status (*n*, %)				0.01
Independent	57 (70.4)	20 (95.2)	37 (61.7)	
Single point stick	6 (7.4)	0 (0)	6 (10.0)	
Wheel walker/wheelchair	18 (22.2)	1 (4.8)	17 (28.3)	
Dialysis treatment				
Access for dialysis (*n*, %)				0.57
Arteriovenous fistula (AVF)	62 (76.5)	16 (76.2)	46 (76.7)	
Tunnelled catheter	15 (18.5)	5 (23.8)	10 (16.7)	
Both AVF and tunnelled catheter	4 (5.0)	0 (0)	4 (6.6)	
Duration of dialysis in years (median, IQR)	2.7 (0.6–6.0)	2.5 (0.7–4.7)	3.1 (0.6–6.4)	0.70
Waitlisting for kidney transplantation (*n*, %)				0.09
Unsuitable	63 (77.8)	13 (61.9)	50 (83.3)	
Under evaluation	12 (14.8)	6 (28.6)	6 (10.0)	
Waitlisted	6 (7.4)	2 (9.5)	4 (6.7)	
**Laboratory parameters**				
Serum creatinine (μmol/L)	618.7 ± 176.2	682.3 ± 142.5	596.4 ± 182.4	0.05
Serum calcium, (mmol/L)	2.4 ± 0.2	2.4 ± 0.1	2.4 ± 0.2	0.84
Serum phosphate (mmol/L)	1.7 (1.4–2.0)	1.7 (1.4–1.9)	1.7 (1.4–2.0)	0.72
Serum parathyroid hormone (pmol/L)	32.1 (21.2–58.8)	34.2 (23.0–60.0)	31.7 (17.0–58.7)	0.51
25(OH)‐vitamin D (nmol/L)	80.0 ± 40.7	75.0 ± 41.3	81.8 ± 40.7	0.51
iFGF23 (pg/mL)	331.3 (156.4–992.2)	1200.0 (156.9–1802.0)	303.7 (154.4–673.7)	0.09
Log (iFGF23)	2.6 ± 0.6	2.8 ± 0.6	2.5 ± 0.6	0.10
cFGF23 (RU/mL)	896.9 (414.9–1702.0)	1012.0 (791.0–2067.0)	690.2 (382.3–1545.0)	0.26
Log (cFGF23)	3.0 ± 0.5	3.0 ± 0.4	3.0 ± 0.5	0.52
Anthropometric and functional performance				
Body mass index in kg/m^2^ (mean, sd)	26.6 ± 5.2	27.5 ± 5.6	26.3 ± 5.0	0.38
Midupper arm circumference in cm (mean, sd)	34.3 ± 4.2	35.5 ± 3.8	33.9 ± 4.3	0.13
Calf circumference in cm (median, IQR)	39.5 (38.1–42.9)	39.8 (37.9–43.6)	39.5 (38.1–42.9)	0.62
Appendicular skeletal muscle index in kg/m^2^ (median, IQR)	6.7 (6.0–7.6)	6.9 (6.0–7.3)	6.7 (6.0–7.7)	0.83
Handgrip strength in kg (median, IQR)	22.0 (16.5–27.5)	30.0 (23.5–36.0)	21.0 (16.0–24.8)	< 0.01
Timed up‐and‐go test in sec (median, IQR)	10.3 (8.4–14.2)	8.3 (6.7–9.9)	11.9 (9.3–15.3)	< 0.01
Chair‐to‐stand test in sec (median, IQR)	14.8 (11.3–18.9)	11.0 (9.2–13.3)	17.0 (14.5–19.7)	< 0.01

*Note:* Values are reported as *N* (%), mean ± SD, or median (IQR).

Abbreviations: AVF, arteriovenous fistula; cFGF23, c‐terminal fibroblast growth factor 23; iFGF23, intact fibroblast growth factor 23; IQR, interquartile range; SD, standard deviation.

**FIGURE 1:**
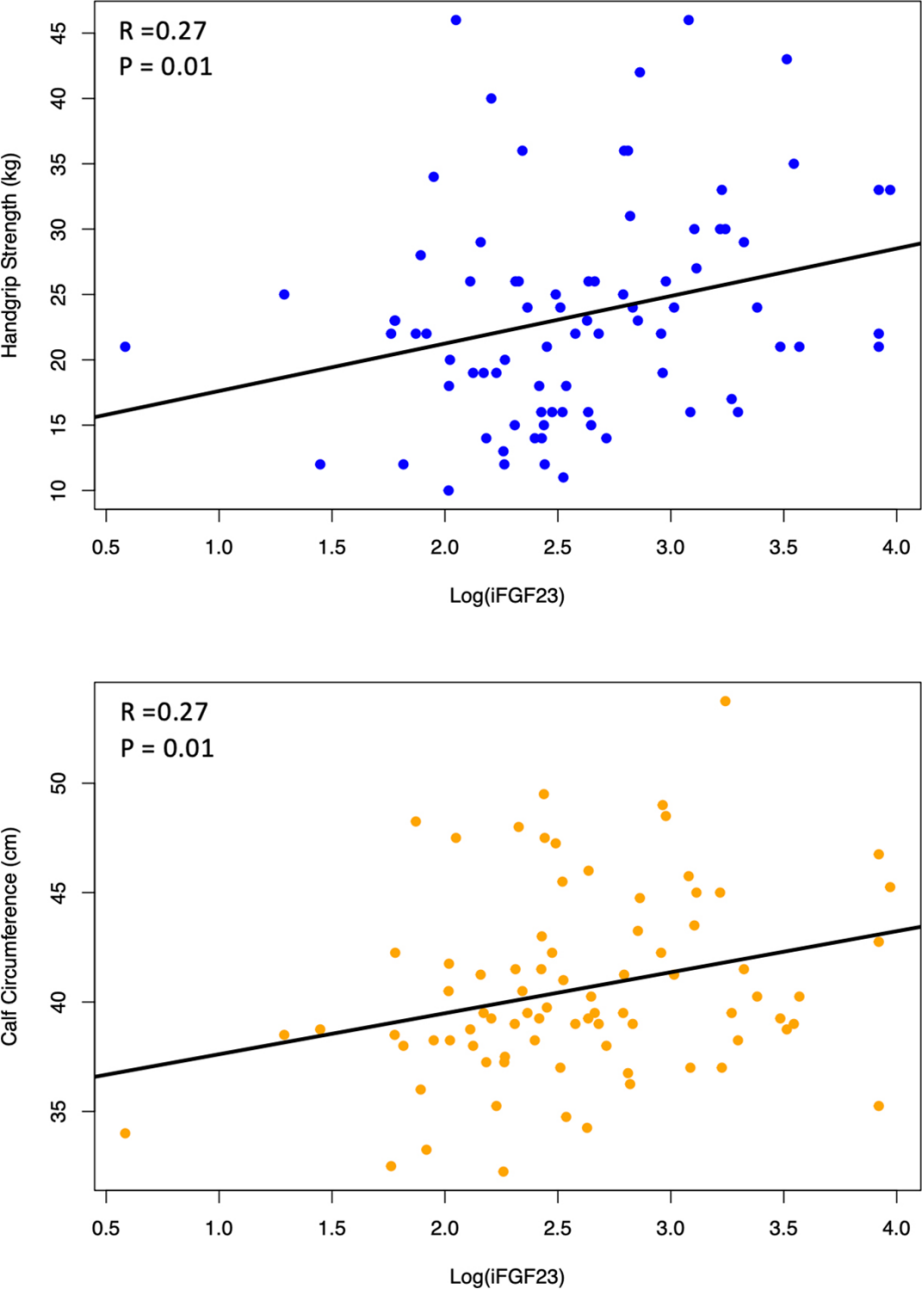
Scatter plots with linear fitted curves and Pearson correlation coefficients between muscle‐related parameters and log‐transformed intact FGF23.

Elevated FGF23 is associated with muscle‐related parameters. We next investigated the potential associations between serum FGF23, laboratory markers and muscle‐related parameters. Significant positive correlations were identified between log‐transformed iFGF23 (log[iFGF23]) with handgrip strength, CC, serum creatinine, urea, phosphate and PTH. Conversely, log (iFGF23) was negatively correlated with age, serum bicarbonate and 25(OH)‐vitamin D. Similar findings were found for log (cFGF23) though the correlation coefficients and *p* values were less significant (Table 2 and Figure 1). We, therefore, focussed on log (iFGF23) for the subsequent analyses. Multiple regression analysis demonstrated that a higher log (iFGF23) was independently associated with greater handgrip strength (beta coefficient [β] = 5.39, 95% CI 2.07–8.72) and conferred a lower risk of sarcopenia (odds ratio (OR) = 0.14, 95% CI 0.02–0.75), after adjustment for age, gender, CCI, BMI, serum creatinine, calcium, phosphate, PTH, 25(OH)‐vitamin D, albumin, IS and CRP (Table 3). In the unadjusted model, a higher log (iFGF23) was associated with greater CC (β = 1.88, 95% CI 0.40–3.35); however, this association was attenuated after correcting for covariates. In contrast, the inverse relationship between log (iFGF23) and sarcopenia was strengthened in the full multivariable model (OR = 0.14, 95% CI 0.02–0.75). Younger age, male sex and lower serum phosphate were independently associated with stronger handgrip strength, whilst higher serum phosphate levels conferred an increased risk of sarcopenia (Tables S1–S3).

**TABLE 2:** Correlations between log‐transformed FGF23 and clinical variables.

Variables	Log (iFGF23)			Log (cFGF23)		
	Correlation coefficient	95% CI	*p*	Correlation coefficient	95% CI	*p*
Age	−0.24	−0.43 to −0.02	0.03	−0.14	−0.35 to 0.08	0.20
CCI	−0.20	−0.40 to 0.03	0.08	−0.13	−0.34 to 0.09	0.25
Urea	0.26	0.04–0.45	0.02	0.09	−0.13 to 0.30	0.44
Serum creatinine	0.37	0.16–0.54	< 0.01	0.39	0.19–0.56	< 0.01
Calcium	0.13	−0.09 to 0.34	0.26	0.23	0.01–0.43	0.04
* Serum phosphate	0.55	0.37–0.69	< 0.01	0.38	0.17–0.56	< 0.01
25(OH) vitamin D	−0.28	−0.47 to −0.07	0.01	−0.27	−0.46 to −0.06	0.01
* Parathyroid hormone	0.27	0.05–0.47	0.01	0.24	0.01–0.44	0.03
Serum bicarbonate	−0.31	−0.50 to −0.10	< 0.01	−0.22	−0.42 to −0.00	0.05
Serum ferritin	−0.08	−0.29 to 0.15	0.50	−0.29	−0.48 to −0.08	< 0.01
Iron saturation	−0.14	−0.34 to 0.09	0.23	−0.33	−0.51 to −0.12	< 0.01
Handgrip strength	0.27	0.06–0.46	0.01	0.17	−0.05 to 0.38	0.12
Calf circumference	0.27	0.06–0.46	0.01	0.22	0.01–0.42	0.04
ASMI	0.05	−0.18 to 0.28	0.67	−0.02	−0.24 to 0.21	0.89
* Timed up‐and‐go test	−0.20	−0.41 to 0.02	0.07	−0.12	−0.34 to 0.11	0.28
Chair‐to‐stand test	−0.06	−0.30 to 0.19	0.65	−0.06	−0.30 to 0.19	0.63

* Correlation between continuous variables was analysed using Spearman's rank correlation coefficients.

Abbreviations: ASMI, appendicular skeletal muscle index; CCI, Charlson Comorbidity Index; CI, confidence interval; log (cFGF23), log‐transformed c‐terminal fibroblast growth factor 23; log (iFGF23), log‐transformed intact fibroblast growth factor 23.

**TABLE 3:** Association between log‐transformed intact fibroblast growth factor 23 (as independent variable) and muscle‐related parameters.

Outcome variable	Predictor variable(s)	β/OR*	95% CI	*p*
Handgrip strength	Unadjusted Model 1 Model 2	3.63 4.59 5.39	0.77–6.50 1.37–7.80 2.07–8.72	0.01 < 0.01 < 0.01
Calf circumference	Unadjusted Model 1 Model 2	1.88 1.56 0.98	0.40–3.35 0.09–3.03 −0.50 to 2.46	0.01 0.04 0.19
Sarcopenia	Unadjusted Model 1 Model 2	0.50* 0.25* 0.14*	0.20–1.13 0.05–1.09 0.02–0.75	0.11 0.08 0.03

*Note:* Model 1 adjusted for age, gender, Charlson Comorbidity Index, body mass index, serum creatinine, calcium, phosphate, parathyroid hormone and 25(OH)‐vitamin D. Model 2 adjusted for covariables in Model 1 plus serum albumin, C‐reactive protein and indoxyl sulphate.

Abbreviations: β, beta coefficient; CI, confidence interval; OR, odds ratio.

**FIGURE 2:**
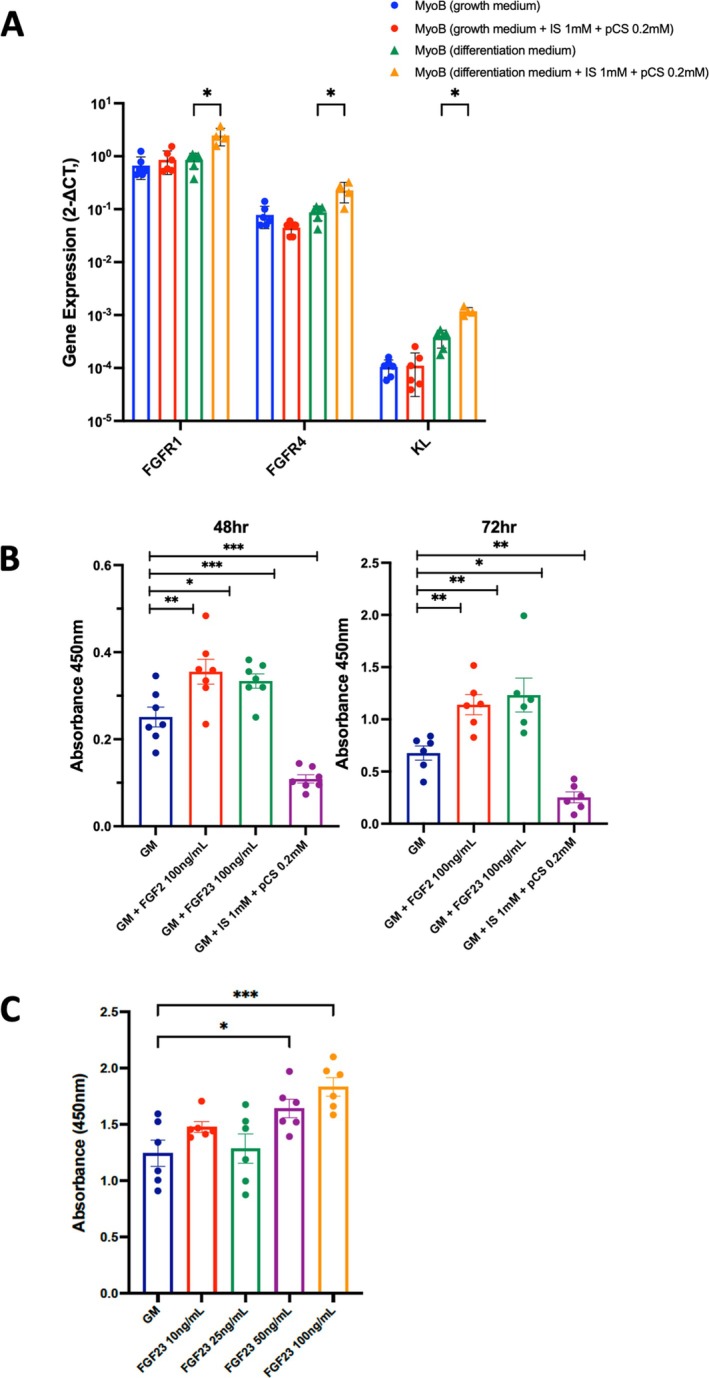
(A) Summary data showing average 2^−∆CT^ value of *FGFR1*, *FGFR4* and *KL* expressed relative to *ACTB* on log10 scale in skeletal muscle myoblasts that were cultured in growth medium (GM) or differentiation medium (DM), complemented or not with indoxyl sulphate (IS) and p‐cresyl sulphate (PCS). Data are mean ± SEM, *n* = 4–6. (B) Cellular proliferation of undifferentiated myoblasts treated with positive control FGF2 (100 ng/mL), FGF23 (100 ng/mL) and negative control uraemic toxins (IS 1 mM + PCS 0.2 mM) for 48 and 72 h, as assessed using BrdU assay. (C) A dose–response experiment with different concentrations of FGF23 (between 10 ng/mL and 100 ng/mL). Data are represented as mean ± SEM, *n* = 6.

**FIGURE 3:**
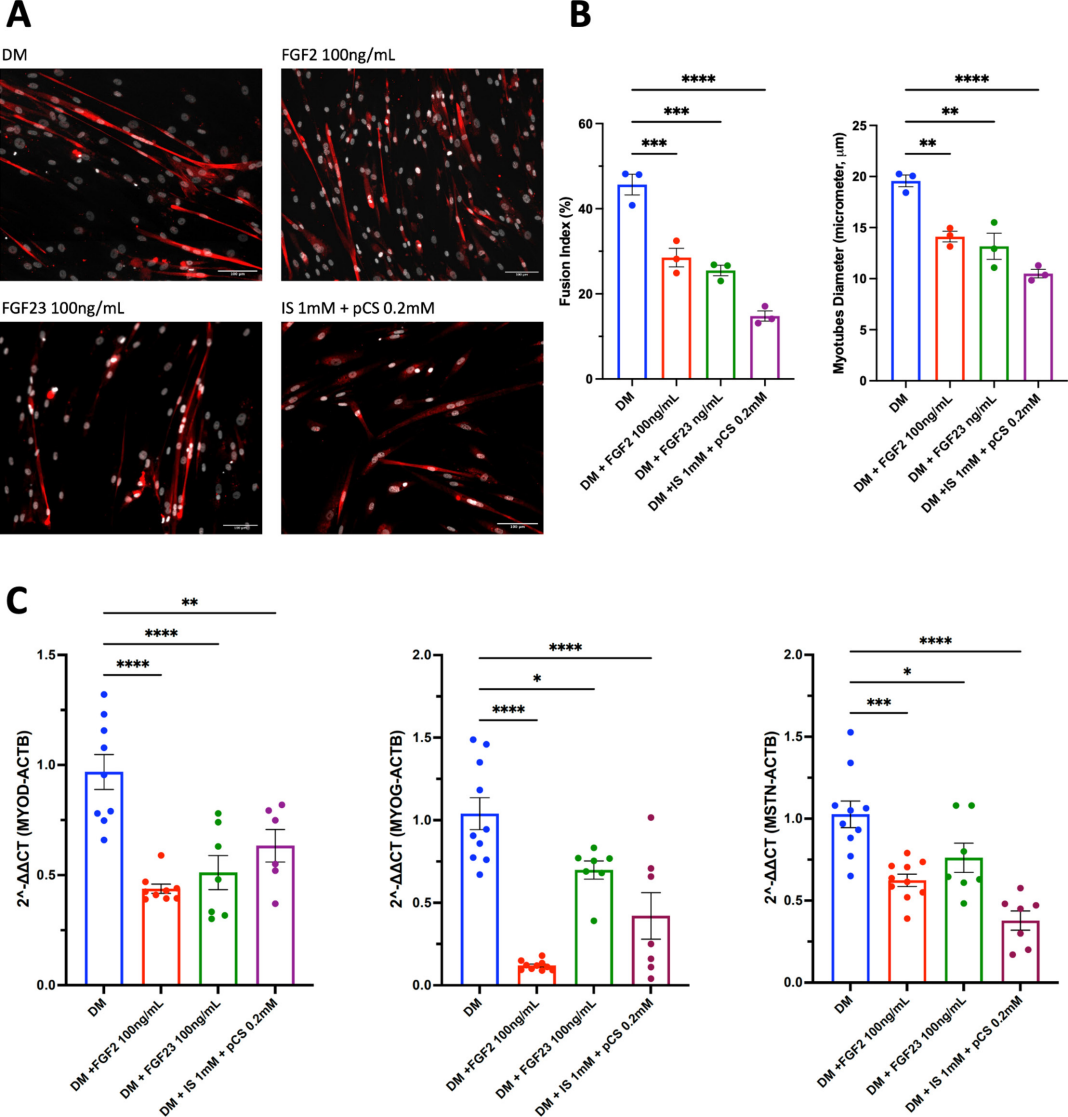
(A) Myogenic differentiation was established using immunofluorescence staining directed against DAPI (white staining) and myosin heavy chain (red staining; representative images *n* = 3 for each condition). (B) Fusion indices and myotubes diameter were calculated at Day 7 of differentiation. (C) The expressions of *MYOD*, *MYOG* and *MSTN* were reduced in myoblasts cultured in differentiated medium (DM) complemented with FGF2, FGF23 and uraemic toxins (*n* = 6–9, data are expressed in mean ± SE).

FGF23 enhances myoblast proliferation but represses myogenic differentiation. As FGFs exert their activity through interaction with FGFR1–4, we first determined their expression in HSMMs and myotubes. We found the expressions of *FGFR1*, *FGFR4* and *a‐klotho* in HSMMs and myotubes in descending order, irrespective of culture conditions (Figure 2A). Low or undetectable expression of *FGFR2* and *FGFR3* was found in those samples tested (data not shown). These findings were comparable to the publicly available tissue‐specific RNA‐sequencing data from the Genotype‐Tissue Expression portal (Figure S1). In addition, the expressions of *FGFR1*, *FGFR4* and *a‐klotho* in myotubes were increased upon exposure to uraemic toxins. *FGFR1* and *FGFR4* are known to express during skeletal muscle development and myogenesis; we therefore investigated the effects of FGF23 on cellular growth pathways. We found a significant increase in cell proliferation in HSMMs treated with recombinant FGF23 (100 ng/mL) at 48 and 72 h. The positive control recombinant FGF2 induced proliferation comparable to previous observations [23, 24], whilst the negative control uraemic toxins had the opposite effect (Figure 2B). A dose‐response experiment was subsequently performed, and an increase in cell proliferation was observed in myoblasts treated with recombinant FGF23 50 ng/mL (Figure 2C) for 48 h. We next assessed the effect of FGF23 on myogenic differentiation. Representative immunofluorescence images of myotubes at Day 7 of differentiation are shown in Figure 3A. The fusion indices were significantly reduced in the presence of FGF2, FGF23, and uraemic toxins, with mean values of 28.5 ± 3.8, 25.5 ± 2.1 and 14.8 ± 2.1, respectively, compared with controls (45.7 ± 4.2). The diameter of myotubes was also significantly decreased (Figure 3B). After 48 h of differentiation, the expressions of myogenic regulatory factors (*MYOD* and *MYOG*) were significantly lower in cells treated with FGF2, FGF23 and uraemic toxins. The expression of myostatin (*MSTN*), a negative regulator of muscle growth and mass, was reduced in cells treated with FGF2, FGF23 and uraemic toxins compared to controls (Figure 3C). Taken together, these findings demonstrate that whilst FGF23 increases skeletal muscle myoblast proliferation, it suppresses myogenic differentiation.

## 4 Discussion

Maintenance dialysis patients with higher circulating iFGF23 levels had stronger handgrip strength and were less likely to be sarcopenic. These findings are in accordance with a previous report by Fukasawa et al. [18], which found positive associations between plasma iFGF23 and computed tomography (CT)‐measured muscle mass and creatinine production rate in haemodialysis patients after adjusting for protein catabolic rate. This supports the view that iFGF23 is associated with muscle mass, independent of dietary protein intake. We observed the strongest association between FGF23 and handgrip strength, whilst the effect for CC was attenuated following adjustment of potential confounding variables. Muscle strength is currently the most reliable measure of muscle function. In the most recent revision of the EWGSOP 2019 guidelines, muscle strength is preferred over mass as it better predicts adverse outcomes [1]. There is clinical precedence for this. Handgrip strength has consistently been shown to be inversely associated with morbidity and mortality in CKD patients [25, 26, 27]. The working group also acknowledged the limitations of current technologies for accurately assessing muscle quantity and quality. Of those recognised measures which can assess muscle mass, CT and magnetic resonance imaging are not widely adopted in clinical practice. In addition, although BIA and dual‐energy X‐ray absorptiometry (DEXA) are more readily available, their reliability may be affected by the fluctuating fluid status exhibited by many CKD patients [10], potentially explaining the lack of association observed between iFGF23 and ASMI in our study.

Another significant finding of the study was the direct effect of FGF23 on myoblast proliferation and differentiation. FGF23 belongs to a heterogeneous superfamily of FGFs that is well recognised to regulate a wide range of cellular functions, including migration, proliferation, differentiation and survival [28]. FGFs mediate many of their biological effects by binding to cell surface receptors, and the expression of FGFRs was developmentally regulated in skeletal muscle [29]. Amongst the four FGFRs, we showed that skeletal muscle myoblasts and myotubes predominantly expressed *FGFR1* and *FGFR4*, both of which were upregulated upon exposure to uraemic toxins. This observation is compatible with previous studies, supporting their roles in myogenesis [30, 31, 32]. We demonstrated that FGF23 enhanced myoblast proliferation but attenuated myogenic differentiation, similar to FGF2 [17, 23, 28], thereby supporting the expansion of the proliferative myogenic pool. FGF23 has previously been shown to inhibit myogenic differentiation through downregulation of insulin and insulin‐like growth factor‐1 signalling [33]. However, this was only published as an abstract. To further substantiate the potential role of FGF23 in muscle development, Shimada et al. demonstrated that targeted ablation of *Fgf23* resulted in skeletal muscle atrophy in homozygous mice, raising the possibility that FGF23 might affect skeletal muscle development and myogenesis in vivo [19], although the observed phenotype could potentially have been confounded by concurrent hyperphosphataemia, which can itself induce cellular senescence and reduce myoblast proliferation [34]. Hyperphosphataemia was independently associated with worsening muscle‐related parameters in our study. Past studies including other models have proven discordant. Chronic exercise was shown to increase FGF23 production in the skeletal muscle of C57BL/6 J mice, and exogenous FGF23 treatment improved exercise performance by controlling the excess production of reactive oxygen species and enhancing mitochondrial function [35]. Yet, Avin et al. showed that FGF23 did not directly influence C2C12 myoblast proliferation and differentiation [36]. The reasons for the conflicting results are unclear but could possibly be explained by the different in vitro models, as significant heterogeneity in the transcriptomic and metabolic profiles was identified between rat L6, mouse C2C12 and primary human skeletal muscle cells [37].

The results from this study have clinical applicability to health professionals caring for patients with CKD. Firstly, over two‐thirds of maintenance dialysis patients in our study had low muscle strength, the key characteristic of sarcopenia with a strong prognostic value. This finding is consistent with a recent meta‐analysis by Duarte et al. [8], highlighting the importance of screening for sarcopenia in maintenance dialysis patients and offering multifaceted interventions to individuals who are at high risk of functional decline, both of which are often not readily accessible in the clinical setting. Moreover, we identified a significant positive association between FGF23 levels and handgrip strength, suggesting that FGF23 could potentially serve as a serum biomarker for sarcopenia in dialysis patients.

Our study has several limitations. Firstly, the generalisability of our findings is not established due to a relatively small sample size in a single‐centre cohort study. Second, the accuracy of muscle mass estimation by BIA might possibly be influenced by fluid status in dialysis patients despite adopting different measures to improve the reliability of measurements. Of note, accurate determination of volume status in dialysis patients remains a significant challenge for all nephrologists. Third, we did not measure soluble α‐klotho. FGF23 regulates mineral homeostasis by activating FGFRs and transmembrane α‐klotho coreceptors. CKD is a state of excess FGF23 and α‐klotho deficiency. High FGF23 levels can act through FGFR4 to promote pathological left ventricular hypertrophy independent of α‐klotho. Given minuscule levels of serum α‐klotho in dialysis patients, we believe the effect of FGF23 on skeletal muscle is likely to be independent of α‐klotho. This finding is substantiated by a previous study [18], where no correlations were identified between muscle mass indices and serum α‐klotho.

In conclusion, sarcopenia is highly prevalent in the maintenance dialysis population. Elevated serum FGF23 was significantly associated with stronger handgrip strength and a lower incidence of sarcopenia. Taken together with our in vitro findings, we postulate that supraphysiological FGF23 concentrations might enhance muscle regeneration by promoting myoblast proliferation. Early sarcopenia screening and timely implementation of interventions are essential to improve the health outcomes of people with CKD, particularly those on dialysis. FGF23 could potentially serve as a serum biomarker of muscle health in patients on dialysis.

## Conflicts of Interest

The authors declare no conflicts of interest.

## Supporting information


**Table S1.** Multiple linear regression models on handgrip strength.
Table S2. Multiple linear regression models on calf circumference.
Table S3. Multiple logistic regression models on sarcopenia.
**Figure S1.** Heatmap of publicly available tissue‐specific gene expression and regulation data downloaded from genotype‐tissue expression portal.
