# Supplementary material for: Fibroblast Growth Factor 23 and Sarcopenia in Maintenance Haemodialysis Population
Source: J Cachexia Sarcopenia Muscle. 2025 Jun 4;16(3):e13848. doi: 10.1002/jcsm.13848 (PMC12134765; doi:10.1002/jcsm.13848)
Supplement: Supplementary file 1 — Table S1. Multiple linear regression models on handgrip strength. Table S2. Multiple linear regression models on calf circumference. Table S3. Multiple logistic regression models on sarcopenia. Figure S1. Heatmap of publicly available tissue‐specific gene expression and regulation data downloaded from genotype‐tissue expression portal. [file JCSM-16-e13848-s001.docx]

**Fibroblast growth factor 23 and sarcopenia in maintenance haemodialysis population**

Limy Wong^1,2^, Rachel Kenny^1^, Jenny YY Ooi^1^, Yung Shing Tsang^1^, Emily Schembri^3^, Lawrence P. McMahon^1,2^

^1^Department of Renal Medicine, Monash University Eastern Health Clinical School, VIC, Australia

^2^Department of Renal Medicine, Eastern Health, VIC, Australia

^3^Monash University Eastern Health Clinical School, VIC, Australia

**Supplementary Methods**

**Supplementary Tables**

Supplementary Table S1. Multiple linear regression models on handgrip strength.

Supplementary Table S2. Multiple linear regression models on calf circumference.

Supplementary Table S3. Multiple logistic regression models on sarcopenia.

**Supplementary Figures**

Supplementary Figure S1. Heatmap of publicly available tissue-specific gene expression and regulation data downloaded from Genotype-Tissue Expression portal.

**Supplementary References**

**Supplementary Methods**

**Human study**

***Human study population***

| **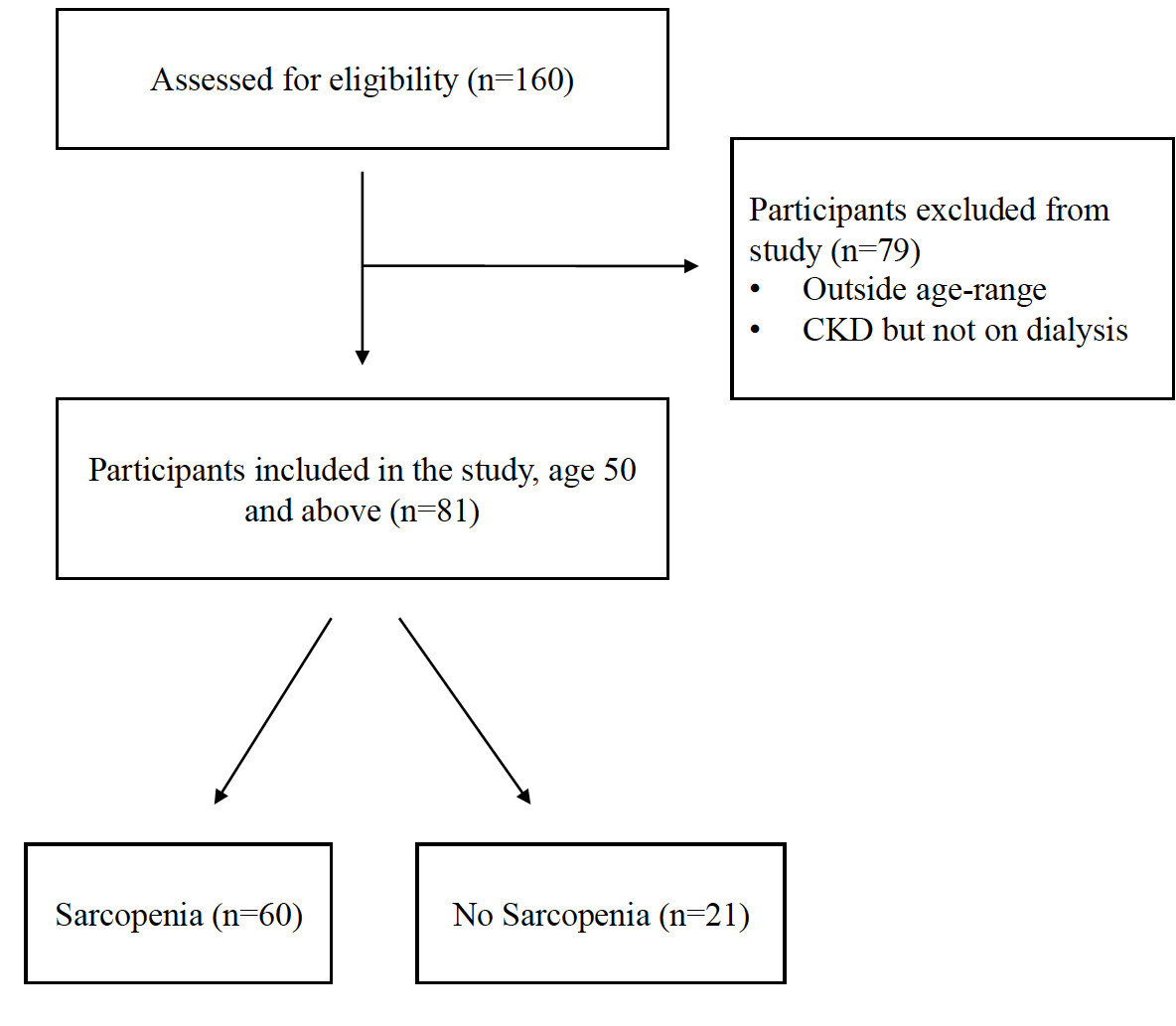** |
| --- |

Flow chart depicting the cress-sectional study.

***Assessment of sarcopenia***

Sarcopenia was defined according both to the revised European Working Group on Sarcopenia in Older People (EWGSOP 2019) and the Asian Working Group for Sarcopenia (AWGS 2019) definition criteria (S1, S2) as a substantial proportion of the study participants were Asian. Three diagnostic categories were defined: the presence of low muscle strength alone (probable sarcopenia), low strength and reduced muscle mass (sarcopenia), and low physical performance (severe sarcopenia). Appendicular skeletal muscle index (ASMI, in kg/m^2^) was derived from the appendicular muscle mass (kg) divided by height squared (m^2^). Muscle mass was measured by bioelectrical impedance analysis (BIA) using a Tanita MC780 Professional Body Composition Scale. Appendicular muscle mass was then calculated using cross-validated BIA Sergi and Yoshida prediction equations for both Caucasian and Asian populations respectively (S3, S4), which are related to the nature of the population in which the equations were developed.

Sergi equation (1):

ASM (kg) = -3.964 + (0.227*RI) + (0.095*weight) + (1.384*sex) + (0.064*Xc)

RI = resistance index (height in centimetres squared/Rz); Sex = female (0), male (1); Xc = reactance

Yoshida equation (2):

Men: ASM (kg) = 0.197 x (impedance index) + 0.179 x (weight) – 0.019

Women: ASM (kg) = 0.221 x (impedance index) + 0.117 x (weight) + 0.881

Impedance index = Height^2^/resistance (measured at 50kHz)

Muscle strength was measured using a Jamar J00105 hydraulic hand dynamometer. Isometric grip force was assessed in both right and left arms, with the participant seated upright with their elbow by their side and flexed at 90° such that their forearm was facing forwards. The highest value was expressed in absolute unit (kg) and used in subsequent analyses. Physical performance was assessed using the Timed-Up and Go test (TUG) where individuals were asked to rise from a standard chair, walk to a marker 3 meters away, turn around, return, and sit down again. All participants were also asked to perform the chair-to-stand test (CST), where the amount of time needed for a participant to rise five times from a seated position without using his or her arms were measured. As the CST requires both strength and endurance, this test is a convenient measure of both muscle strength and physical performance.

Criteria for categorising participants as sarcopenic:

| **Parameter** | **EWGSOP 2019 (S2)** | **AWGS 2019 (S1)** |
| --- | --- | --- |
| Muscle mass  ASM/height^2^ (using bioelectrical impedance analysis) | Men <7.0kg/m^2^  Women <5.5kg/m^2^ | Men <7.0kg/m^2^  Women <5.7kg/m^2^ |
| Muscle strength  Handgrip strength using hand dynamometer | Men <27kg  Women <16kg | Men <28kg  Women <18kg |
| Physical performance  ^†^Timed-Up and Go test OR  ^‡^Five time chair-to-stand test | ≥20s  >15s | ≥20s  >12s |

^†^Based on the AWGS 2019, timed-up-and-go (TUG) is not recommended, because the results might reflect multiple complex pathoetiologies while five time chair stand test is included as one of recommended physical performance measurement that has been found to predict mortality.

^‡^A 5-time chair-to-stand time cut-off of 11.6 seconds corresponded to a walking speed of 1.0m/s; hence, AWGS 2019 recommends ≥12 seconds as the cut-off for low physical performance.

**Experimental studies**

***Real-time polymerase chain reaction***

A list of the TaqMan^®^ Gene Expression Assays:

| **Gene** | **Assay ID** | **Amplicon length** |
| --- | --- | --- |
| FGFR1 | Hs00241111_m1 | 81 |
| FGFR2 | Hs01552918_m1 | 74 |
| FGFR3 | Hs00179829_m1 | 104 |
| FGFR4 | Hs01106910_g1 | 90 |
| KL | Hs00934627_m1 | 108 |
| MYOD | Hs00159528_m1 | 67 |
| MYOG | Hs01072232_m1 | 76 |
| MSTN | Hs00976237_m1 | 69 |
| ACTB | Hs99999903_m1 | 171 |

**Supplementary Tables**

**Supplementary Table S1. Multiple linear regression models on handgrip strength.**

| **Variable** | **Unadjusted** | | | **Model 1** | | | **Model 2** | | |
| --- | --- | --- | --- | --- | --- | --- | --- | --- | --- |
|  | **Estimate** | **P value** | **95% CI** | **Estimate** | **P value** | **95% CI** | **Estimate** | **P value** | **95% CI** |
| Log(iFGF23) | 3.63 | 0.01 | 0.77 – 6.50 | 4.59 | <0.01 | 1.37 – 7.80 | 5.39 | <0.01 | 2.07 – 8.72 |
| Age (in years) |  |  |  | -0.27 | 0.01 | -0.48 – -0.06 | -0.24 | 0.03 | -0.46 – -0.03 |
| Gender (female) |  |  |  | -7.11 | <0.01 | -10.15 – -4.06 | -6.85 | <0.01 | -9.88 – -3.81 |
| CCI |  |  |  | -0.53 | 0.18 | -1.32 – 0.25 | -0.58 | 0.15 | -1.38 – 0.21 |
| BMI (in kg/m2) |  |  |  | 0.13 | 0.37 | -0.16 – 0.42 | 0.19 | 0.21 | -0.11 – 0.48 |
| Creatinine |  |  |  | 0.002 | 0.72 | -0.01 – 0.01 | 0.003 | 0.56 | -0.01 – 0.01 |
| Calcium |  |  |  | -6.09 | 0.20 | -15.51 – 3.33 | -7.89 | 0.11 | -17.57 – 1.79 |
| Phosphate |  |  |  | -3.41 | 0.03 | -6.44 – -0.38 | -3.95 | 0.01 | -7.01 – -0.89 |
| PTH |  |  |  | -0.04 | 0.04 | -0.08 – -0.00 | -0.04 | 0.06 | -0.08 – 0.00 |
| Vitamin D |  |  |  | 0.002 | 0.91 | -0.03 – 0.04 | 0.005 | 0.77 | -0.03 – 0.04 |
| Albumin |  |  |  |  |  |  | -0.26 | 0.29 | -0.74 – 0.23 |
| Indoxyl Sulphate |  |  |  |  |  |  | -0.07 | 0.40 | -0.23 – 0.09 |
| CRP |  |  |  |  |  |  | -0.20 | 0.08 | -0.43 – 0.03 |

Abbreviations: BMI, body mass index; CCI, Charlson Comorbidity Index; CI, confidence interval; CRP, C-reactive protein; log(iFGF23), log-transformed intact fibroblast growth factor 23; PTH, parathyroid hormone.

**Supplementary Table S2. Multiple linear regression models on calf circumference.**

| **Variable** | **Unadjusted** | | | **Model 1** | | | **Model 2** | | |
| --- | --- | --- | --- | --- | --- | --- | --- | --- | --- |
|  | **Estimate** | **P value** | **95% CI** | **Estimate** | **P value** | **95% CI** | **Estimate** | **P value** | **95% CI** |
| Log(iFGF23) | 1.88 | 0.01 | 0.40 – 3.35 | 1.56 | 0.04 | 0.09 – 3.03 | 0.98 | 0.19 | -0.50 – 2.46 |
| Age (in years) |  |  |  | -0.07 | 0.18 | -0.16 – 0.03 | -0.06 | 0.25 | -0.15 – 0.04 |
| Gender (female) |  |  |  | 0.34 | 0.63 | -1.05 – 1.73 | 0.26 | 0.70 | -1.09 – 1.61 |
| CCI |  |  |  | 0.13 | 0.49 | -0.23 – 0.48 | 0.08 | 0.65 | -0.27 – 0.44 |
| BMI (in kg/m2) |  |  |  | 0.59 | <0.01 | 0.46 – 0.72 | 0.58 | <0.01 | 0.45 – 0.71 |
| Creatinine |  |  |  | 0.001 | 0.77 | -0.00 – 0.00 | 0.002 | 0.44 | -0.00 – 0.01 |
| Calcium |  |  |  | -1.02 | 0.64 | -5.33 – 3.28 | 0.49 | 0.82 | -3.82 – 4.81 |
| Phosphate |  |  |  | -0.003 | 0.99 | -1.39 – 1.38 | 0.28 | 0.68 | -1.08 – 1.65 |
| PTH |  |  |  | -0.01 | 0.55 | -0.02 – 0.01 | -0.01 | 0.40 | -0.03 – 0.01 |
| Vitamin D |  |  |  | 0.01 | 0.37 | -0.01 – 0.02 | 0.01 | 0.49 | -0.01 – 0.02 |
| Albumin |  |  |  |  |  |  | 0.02 | 0.85 | -0.20 – 0.24 |
| Indoxyl Sulphate |  |  |  |  |  |  | -0.01 | 0.86 | -0.08 – 0.07 |
| CRP |  |  |  |  |  |  | 0.14 | <0.01 | 0.04 – 0.24 |

Abbreviations: BMI, body mass index; CCI, Charlson Comorbidity Index; CI, confidence interval; CRP, C-reactive protein; log(iFGF23), log-transformed intact fibroblast growth factor 23; PTH, parathyroid hormone.

**Supplementary Table S3. Multiple logistic regression models on sarcopenia.**

| **Variable** | **Unadjusted** | | | **Model 1** | | | **Model 2** | | |
| --- | --- | --- | --- | --- | --- | --- | --- | --- | --- |
|  | **OR** | **P value** | **95% CI** | **OR** | **P value** | **95% CI** | **OR** | **P value** | **95% CI** |
| Log(iFGF23) | 0.50 | 0.11 | 0.21 – 1.13 | 0.25 | 0.08 | 0.05 – 1.10 | 0.14 | 0.03 | 0.02 – 0.75 |
| Age (in years) |  |  |  | 1.02 | 0.60 | 0.94 – 1.11 | 1.02 | 0.72 | 0.93 – 1.12 |
| Gender (female) |  |  |  | 0.29 | 0.085 | 0.06 – 1.14 | 0.27 | 0.12 | 0.05 – 1.31 |
| CCI |  |  |  | 1.32 | 0.11 | 0.95 – 1.89 | 1.40 | 0.08 | 0.97 – 2.10 |
| BMI (in kg/m2) |  |  |  | 0.96 | 0.55 | 0.84 – 1.09 | 0.94 | 0.40 | 0.82 – 1.08 |
| Creatinine |  |  |  | 0.99 | 0.24 | 0.99 – 1.00 | 0.99 | 0.69 | 0.99 – 1.00 |
| Calcium |  |  |  | 5.19 | 0.45 | 0.08 – 433.6 | 27.79 | 0.18 | 0.21 – 5328 |
| Phosphate |  |  |  | 5.64 | 0.05 | 1.17 – 39.57 | 6.56 | 0.04 | 1.31 – 53.70 |
| PTH |  |  |  | 0.99 | 0.87 | 0.98 – 1.02 | 0.99 | 0.50 | 0.98 – 1.01 |
| Vitamin D |  |  |  | 0.99 | 0.77 | 0.98 – 1.02 | 0.99 | 0.59 | 0.98 – 1.01 |
| Albumin |  |  |  |  |  |  | 0.97 | 0.83 | 0.75 – 1.25 |
| Indoxyl Sulphate |  |  |  |  |  |  | 1.01 | 0.75 | 0.94 – 1.09 |
| CRP |  |  |  |  |  |  | 1.20 | 0.05 | 1.04 – 1.52 |

Abbreviations: BMI, body mass index; CCI, Charlson Comorbidity Index; CI, confidence interval; CRP, C-reactive protein; log(iFGF23), log-transformed intact fibroblast growth factor 23; PTH, parathyroid hormone.

**Supplementary Figures**

**Supplementary Figure S1 .** (A) Heatmap of publicly available tissue-specific gene expression and regulation data downloaded from Genotype-Tissue Expression portal (<https://gtexportal.org/home/>). (B) *FGFR1*, *FGFR4* and *a-klotho* were expressed in descending order in human skeletal muscle (data were expressed as transcripts per million (TPM), for every 1,000,000 RNA molecules in the RNA-seq sample)

| **A.**  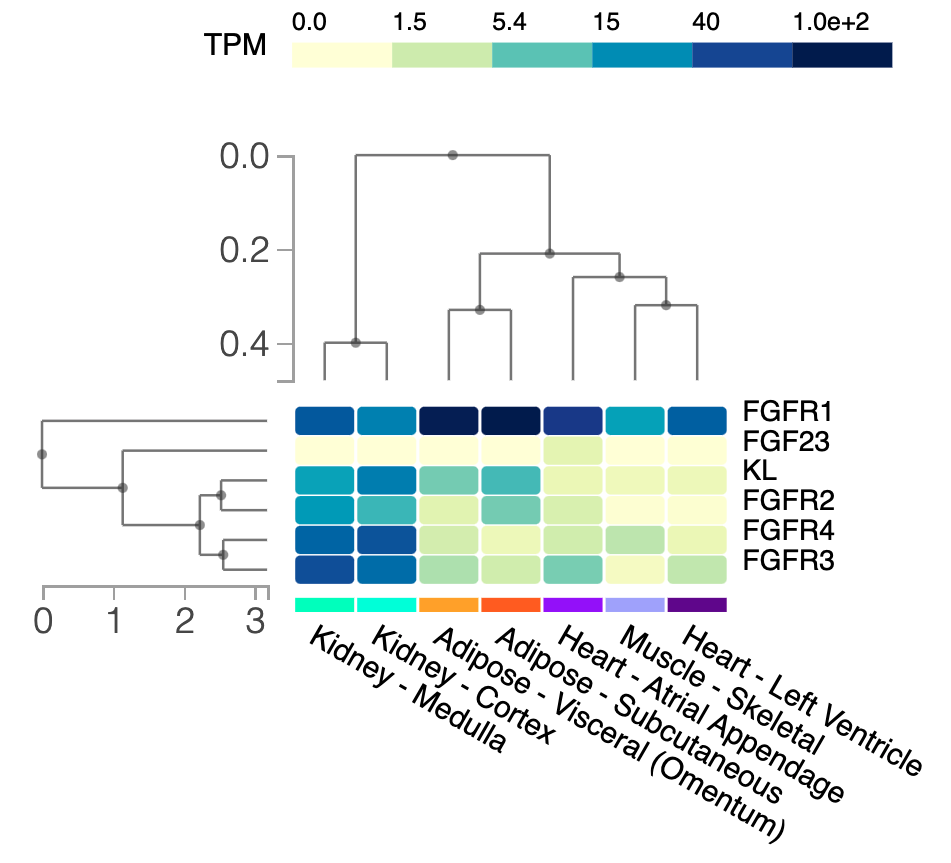 |
| --- |
| **B.**   \| **Gene** \| **Kidney -medulla** \| **Kidney - cortex** \| **Adipose - visceral** \| **Adipose - subcutaneous** \| **Heart – atrial appendage** \| **Skeletal muscle** \| **Heart – left ventricle** \| \| --- \| --- \| --- \| --- \| --- \| --- \| --- \| --- \| \| **FGFR1** \| 30.30 \| 18.03 \| 94.70 \| 102.2 \| 49.81 \| 10.91 \| 27.41 \| \| **FGF23** \| 0 \| 0 \| 0 \| 0 \| 0.8421 \| 0 \| 0.03432 \| \| **KL** \| 10.74 \| 18.73 \| 4.297 \| 6.653 \| 0.6381 \| 0.5112 \| 0.5342 \| \| **FGFR2** \| 12.37 \| 7.724 \| 0.9301 \| 4.287 \| 1.219 \| 0.06111 \| 0.08663 \| \| **FGFR4** \| 25.93 \| 32.59 \| 1.361 \| 0.540 \| 1.430 \| 1.965 \| 0.6379 \| \| **FGFR3** \| 34.57 \| 23.23 \| 2.426 \| 1.426 \| 4.103 \| 0.3384 \| 1.878 \| |

**Supplementary References**

S1. Chen LK, Woo J, Assantachai P, Auyeung TW, Chou MY, Iijima K, et al. Asian Working Group for Sarcopenia: 2019 Consensus Update on Sarcopenia Diagnosis and Treatment. J Am Med Dir Assoc. 2020;21(3):300-7 e2.

S2.         Cruz-Jentoft AJ, Bahat G, Bauer J, Boirie Y, Bruyere O, Cederholm T, et al. Sarcopenia: revised European consensus on definition and diagnosis. Age Ageing. 2019;48(1):16-31.

S3.         Sergi G, De Rui M, Veronese N, Bolzetta F, Berton L, Carraro S, et al. Assessing appendicular skeletal muscle mass with bioelectrical impedance analysis in free-living Caucasian older adults. Clin Nutr. 2015;34(4):667-73.

S4.         Yoshida D, Shimada H, Park H, Anan Y, Ito T, Harada A, et al. Development of an equation for estimating appendicular skeletal muscle mass in Japanese older adults using bioelectrical impedance analysis. Geriatr Gerontol Int. 2014;14(4):851-7.
